# Synthesis, Characterization and Performance of Materials for a Sustainable Future

**DOI:** 10.3390/nano13131929

**Published:** 2023-06-25

**Authors:** John Vakros, Evroula Hapeshi, Catia Cannilla, Giuseppe Bonura

**Affiliations:** 1 Department of Chemical Engineering, University of Patras, Caratheodory 1, University Campus, GR-26504 Patras, Greece; 2 Pharmacy Programme, Department of Health Sciences, School of Life and Health Sciences, University of Nicosia, 46 Makedonitissas Avenue, CY-2417, P.O. Box 24005, CY-1700 Nicosia, Cyprus; hapeshis.e@unic.ac.cy; 3 Institute for Advanced Energy Technologies “Nicola Giordano” ITAE, National Research Council (CNR), 98126 Messina, Italy; catia.cannilla@itae.cnr.it (C.C.); giuseppe.bonura@itae.cnr.it (G.B.)

Today, sustainability represents the key factor for economic progress in compliance with social advancement and environmental protection, driving innovation in materials, processes and technologies. The main seven principles of sustainability are sustainable design, durability, energy efficiency, waste reduction, indoor air quality, water conservation, and sustainable building materials. The sustainability of a process can be highly influenced by changing the material, or replacing an already working material with another. It is evident that the material can influence almost all of the above principles. Therefore, arising from the need for innovative chemical formulations for advanced (nano)materials, inorganic/organic/coordination compounds, porous composites, hybrid/multifunctional or nanostructured supported systems, recent progresses, challenges, and opportunities for the application of functional materials in different research fields are all presented. The different fields include catalysis, photocatalysis, electronics, optics, energy and the environment, and medicine, with their shared significance being that the application of new, greener, sustainable materials will facilitate the development of new products with respect to sustainable growth.

The application of green compounds in different processes and the replacement of traditional solvents is one possible way for a more sustainable future. Eucalyptol, 1,8-cineole, can be used for the treatment of disorders, while additionally it can replace a solvent in the synthesis of O,S,N-heterocycles as discussed in [1], indicating the importance and potential of bio solvents and their viability in synthesis laboratories.

The valorization of wastes to produce useful and high-value materials is always desirable from a technological and economical point of view. In this regard, PET is one of the most commonly present materials in everyday waste, and in this study, [2] recycled PET was used for the production of filter materials using electrohydrodynamic techniques. It was discovered that in this process, the distance between the needle and the collector, the polymer concentration and output flow rate are the most crucial parameters. These results can be used to develop a wide variety of filter media with different applications. One interesting application is the manufacturing of surgical masks by electrospinning.

In medicine, one advanced method of releasing drugs is in situ. Affibody-modified G-quadruplex DNA micelles incorporating polymeric 5-fluorodeoxyuridine were applied for the targeted delivery of drugs [3]. To achieve synergistic efficacy with the hydrophobic drugs, curcumin (Cur) was co-loaded. The affibody was used to facilitate HER2-receptor-mediated uptake. The drug loading rates were 21.1% for FUdR and 5.6% for Cur. The Cur@affi-F/GQs showed a higher cytotoxicity and greater synergistic effect on HER2-positive gastric cancer N87 cells.

The manufacturing of alloys is always a challenge. Hierarchical surface nano-/microstructuring by femtosecond laser micromachining was applied for the preparation of a novel multifunctional material [4]. The hierarchical surface nano-/microstructure developed was an array of nanostructured microgrooves fabricated on the surface of a Ti-6Al-4V alloy substrate. Ti-6Al-4V shows long-term stability at high temperatures, and the wind-tunnel experiments demonstrated its competent and long-lasting wicking and evaporative functionalities under the conditions of high-temperature airflows, indicating its suitability for practical applications.

Nanostructured ferritic (Fe_(82−x)_-Cr_18_-Si_x_, x = 0–3 wt %) and austenitic (Fe_(73−x)_-Cr_18_-Ni_9_-Si_x_, x = 0–3 wt %) stainless steel (SS) alloys were prepared, characterized and tested for their electrochemical performances [5]. The preparation was performed by mechanical alloying and spark plasma sintering, while characterization showed that, with an increase in the wt % of Si content, the unit cell parameter shows a decreasing trend. Additionally, their electrochemical performances increase with Si loading due to densification.

When two prepared surfaces are joined at elevated temperatures, multiple factors affect the diffusion bonding process. Among them, surface roughness, contamination and other factors are responsible for defects. An analysis method for the detectability of defects on the TC4 (Ti-6Al-4V) alloy was presented based on the ultrasonic detectability of interface gap defects and was experimentally verified [6].

A simple and lithography-free structure-replication process to obtain large-scale surface cup-shaped nano-pillar (CSNP) arrays has also been employed [7]. The CSNP nanostructured Si showed an excellent surface anti-reflection performance when compared to that of the single-polished Si. Additionally, it is polarization insensitive. Interestingly, when used in the form of thin film solar cell, an improvement of 54.64% was achieved for the CSNP in comparison to the optimized Si_3_N_4_.

The following are two examples where industrial waste can be used as a raw material for high-value chemicals. In recent work [8], the production of amorphous silica from aluminum-fluoride industrial waste was discussed. As a starting material, a silica gel with a moisture content of up to 55 wt % and a SiO_2_/AlF_3_ ratio of 4 was used. The purification of the starting material was performed through basic and acidic solutions. The characterization techniques demonstrated that the products are of high purity, form agglomerates of spherical SiO_2_ particles up to 1 μm in size and can replace products manufactured by existing technologies, for example, catalyst supports with increased strength.

The generation process, impurity-removal treatment (physical method, chemical method, heat method) and high-value utilization (nanometer calcium sulfate whisker, nanometer calcium carbonate) of the material phosphogypsum were presented and discussed [9], since phosphogypsum constitutes a large amount of solid waste and its high-value valorization could contribute to sustainability.

Another group of materials that can be applied in environmental applications, like the removal of organic contaminants from water matrices, are the Layer Double Hydroxides (LDHs). They have interesting properties and are also among the most promising materials for electrochemical capacitors. LDHs usually contain bivalent and trivalent metal ions like Co(II)/Co(III) and Fe(III)/Fe(II). In the work of Morcos et al. [10], the coprecipitation method was used to prepare Co/Fe-based LDHs. The materials were characterized with different physicochemical techniques, like powder X-ray diffractometry, Fourier Transform Infra-red spectroscopy, Scanning Electron Microscopy, etc. It was found that the crystallinity of the ferrihydrite decreases with an increasing pH. Hydrolysis of both the sorbed and free Co species generates pristine hydroxylated Fe(II)/Co(III) LDHs at pH 7, while at a higher pH the local dissolution of pristine LDHs, re-precipitation, and then 3D organization into Co^II^_4_Fe^II^_2_Co^III^_2_ LDHs occur. The incorporation of Co(II) into the LDH structure decreases coulombic attraction between the positive surface-charge sites and the interlayer anions, and Fe(II) is re-oxidized to Fe(III), while Co(III) is re-reduced to Co(II), returning to a Co^II^_6_Fe^III^_2_ LDH. The adsorption of phosphates on LDHs was studied in [11]. The researchers synthesized glycine- and alanine-intercalated LDHs, characterized them and applied them to phosphate adsorption. It was found that Gly-Cl-LDH and Ala-Cl-LDH had better physicochemical characteristics than Cl-LDH, better adsorption performances at a lower pH and a better adsorption selectivity against SO_4_^2−^. The kinetics of adsorption follow pseudo-second-order constants, and can reach equilibrium in shorter time than Cl-LDH. The maximum adsorption capacities were 63.2 mg-P L^–1^, 55.8 mg-P L^–1^ and 58.2 mg-P L^–1^, for Cl-LDH, Gly-Cl-LDH and Ala-Cl-LDH, respectively.

The adsorption of organic pollutants on the surface of a solid sorbent is a simple method for removing the pollutants from water, but in order to be efficient the solid material must be stable, easy to regenerate and possess a high adsorbance capacity. In [12], a core-sheath structure was designed and prepared via one-step co-axial electrospinning for the fabrication of electrospun cellulose-acetate (CA)/chitosan (CS) adsorbents for humic-acid (HA) removal. The modified preparation lead to fibers with smaller diameters, greater homogeneity, and significantly improved mechanical strength, although their maximum adsorption capacities for HA were similar; however, it is important that the fibers maintained their strength and shape even after the complete hydrolysis of CA. Another example is the modification of nanosilica with amine. This was studied in [13] when it was prepared under very mild conditions, in a water-in-oil microemulsion with a non-ionic surfactant N-[3-(Trimethoxysilyl)propyl]ethylenediamine (DA). The effect of the quantity of DA on the properties of the amine-modified nano-silica was determined using various techniques, and it was discovered that the amount of DA has a strong influence on the properties of the samples, especially on the crystallinity, and, thus, on the value of the surface area. The samples prepared with 1.5 mL of DA (SNP-1.5DA) showed a better adsorption performance compared to the samples prepared with 0.5 and 1.0 mL of DA.

Covalent organic frameworks (COF) are porous crystalline compounds made up of organic material bonded together by strong reversible covalent bonds. These compounds are entirely made up of light elements like H, B, C, N, O and Si, and can be applied to significant applications, like the removal of Naproxen from water through adsorption [14]. Different COFs were prepared, characterized and tested as sorbents. Naproxen removal rates of 70% and 86% were determined for 210 min and 270 min, respectively, at a constant dose of 0.05 g and pH 7. The COFs were stable at least to pH = 9. The maximum adsorption abilities of the substances were found to be 35 mg/g and 42 mg/g while the adsorption was under chemical process control.

When the solid sorbent is a semiconductor then the material can act not only as sorbent but also as a catalyst under light irradiation. Due to the absorption of light, electrons from the semiconductor are moved in the conduction band, while holes with positive charges are created in the valence band. Subsequently, electrons and holes can degrade the organic pollutant. This process is called photocatalysis and is one of the most important processes for the oxidation of organic pollutants in water matrices. In the work of Ghaffari et al. [15], Mn_2_O_3_-Fe_2_O_3_@SiO_2_ was used as a photocatalyst. This catalyst, although active for erythromycin adsorption and degradation, is not stable. On the contrary, the photocatalyst is stable after pre-treatment with UV irradiation. It was reported that the treated catalyst can retain its activity for at least 10 cycles. Extensive physicochemical characterization showed that UV irradiation significantly affects the nanocomposite structure, where SiO_2_ network reconfiguration changes the surface –OH group density and the specific surface area of the photo-catalyst. These modifications can explain the enhanced performance of the treated catalyst. Moreover, the oxidation of adsorbed molecules by UV light after each cycle could be another reason for enhanced removal. For the first time, the fate of erythromycin was studied using regenerated nanocomposites after the last cycle. LC/MS/MS results showed that erythromycin degraded in 20 min, and the produced reaction by-products were adsorbed by nanocomposites.

The degradation of organic contaminants from water is a significant process for sustainability. Instead of adsorption, where the contaminant still exists, catalytic degradation will eliminate the organic pollutant, and photocatalysis, especially with TiO_2_ as the catalyst, is one of the most studied processes for the degradation of organic noxious waste. Mono-doped (M1: Mo, W) and Co-doped (M2-M1: Cu, Co, Zn) titania catalysts were applied for the degradation of 4-tert-butylphenol in water [16]. The samples were prepared with a wet impregnation method, characterized with various physicochemical techniques and tested for the degradation of 4-tert-butylphenol. It was found that Cu-Mo-TiO_2_ showed better catalytic activity than pure TiO_2_, achieving complete degradation of 4-tert-butylphenol under UV-light irradiation after 60 min. The application of Cu-Mo-TiO_2_ under solar light conditions was also tested, and 70% of 4-tert-butylphenol degradation was achieved within 150 min. In another work [17], TiO_2_ samples loaded with Cu at concentrations from 0.1 to 1.0 wt % were prepared and tested as photocatalysts for the degradation of methylene blue (MB) and for the photokilling activity of Escherichia coli (*E. coli*). The sample with the lowest Cu content (i.e., 0.1 wt %) was found to be the most active, with an increase in Cu loading producing a detrimental effect on the performance of the photocatalyst.

Materials with antibacterial performances can be used in food packaging. Poly(lactic acid) (PLA) films containing phloridzin adsorbed on to a MCM-41 mesoporous molecular sieve were prepared using electrostatic spinning in order to be used as food packaging [18]. PLA was the film-forming substrate, phloridzin acted as an antioxidant, and MCM-41 acted as the adsorption and controlled-release carrier. The best results, exemplified by a free-radical scavenging rate as high as 53.61% along with a good antibacterial performance (85.22%), were obtained with a mass ratio of 1:2 (MCM-41:phloridzin). In a real-world application in strawberry packaging, the prepared films significantly delay lipid oxidation up to 21 days before mildew appears.

The selection of the preparation method is significant for the final properties of the material. There are many different synthetic routes and preparation methods, but also almost unlimited ways to modify a given route. Some examples are presented in this editorial concerning biosynthesis, sol-gel method, ball-milling synthesis, plasma-based methods, ultrasonic irradiation, etc.

Biosynthesis can be used for the production of nanomaterials in some cases. In the study of Anees et al. [19], the green synthesis (sunlight exposure method) of Ag nanoparticles from leaf extracts of *Azadirachta indica* and *Pongamia pinnata* was performed. The nanoparticles were tested, which demonstrated their bioefficacy on *H. armigera* (2nd instar). The Ag nanoparticles have an average diameter of 61.70 nm (*A. indica*) and 68.80 nm (*P. pinnata*) and have spherical shape. A. indica-based silver nanoparticles were found to be comparatively more efficient and have higher insecticidal activity compared to *P. pinnata*-based nanoparticles. A. indica-based AgNPs had the maximum negative zeta potential of −58.96 mV and could be stored for three months without losing bioefficacy and up to six months with a negligible reduction in bioefficacy.

A one-pot green method for the aqueous synthesis of fluorescent copper sulfide nanoparticles (NPs) was developed in [20], using Cu(II) and cysteine. The reaction occured at 37 °C, at a physiological pH under aerobic conditions. The prepared nanoparticles exhibited green fluorescence, a size between 8–12 nm, a hexagonal chalcocite crystalline structure and a 3.5 ± 0.1 Å interplanar atomic distance. The experiment successfully demonstrated the performance of biomimetic Cu_2_S NPs as counter electrodes in photovoltaic devices, constructed using different sensitizers and electrolytes.

Another example of using biosynthesis to prepare nanoparticles was the synthesis of platinum nanoparticles using Cordyceps flower extract [21]. An extensive characterization of the nanoparticles revealed that they were spherical particles covered with Cordyceps flower extract, with average particle size of 84.67 ± 5.28 nm (in Dynamic Light Scattering), or 13.34 ± 4.06 nm determined by Transmission Electron Microscopy. The Pt nanoparticles had a significant antioxidant activity. Depending on the bacteria used for the antibacterial activity of the nanoparticles, morphological changes were observed, with stronger antibacterial activity of PtNPs against *Gram-negative* bacteria.

The sol-gel method is another promising preparation method of high value materials. Changing the parameters of the synthesis, the physicochemical properties of the material can be tuned. The influence of different aminoalcohols (AA) on ZnO films was studied [22], and it was found that aliphatic films were more stable and pure than aromatics. The aliphatic films have ZnO in the form of wurtzite, while the aromatic AAs were of a cubic sphalerite (zinc-blende) form. UV and photoluminescence studies revealed that these AAs also affect the optical band gap, structural defects, and photo-optical properties of the films.

Currently, sodium-ion battery cathodes are a hot topic and Prussian blue represents a promising candidate in this field [23], although the interstitial water in its crystal structure and its poor electronic conductivity limit its performance. Surface-modified Prussian blue can be produced with the targeted use of acid-assisted ball-milling synthesis resulting in a higher specific surface area. To reduce the interstitial water concentration, carboxyl functional groups were introduced into Prussian blue. Its application as cathode demonstrated that the material has a specific capacity of 145.3 mAh g^−1^ at 0.2 C and 113.7 mAh g^−1^ at 1 C, and it is quite stable (54.5% after 1000 cycles).

A plasma-enhanced atomic-layer deposition method was applied for doping Ga_2_O_3_ films with Sn at the atomic level [24]. The systematic characterization of the doped films proved that all the films have a high transparency (above 90%). The Sn doping affects the density, refractive index and extinction coefficient of the films. As expected, the ratio of Sn–O bonding increases with Sn content, although the proportion of the oxygen vacancies decreases. Moreover, the breakdown mode transformed from a hard breakdown into a soft breakdown, and the Sn-doped Ga_2_O_3_ films had a large permittivity.

In the work of Hwangbo et al. [25], an efficient method for surfactant-free nano-emulsification using ultrasound was presented. The comparison of three surfactant-free ultrasonic emulsification technologies (horn, bath and focused ultrasonic systems) was presented, and the focused ultrasonic system exhibited the best results. This was due to the fact that this configuration concentrates sound energy at the center of the dispersion system. The emulsion has a particle size distribution of 60–400 nm at 400 kHz and no phase separation was observed. The absence of surfactants in this eco-friendly emulsification process increases its sustainability.

A recent focus of investigation is the production of superhydrophobic fluorine-free and biodegradable materials for oil/water separation. Two biopolymer oil/water separation routes in cellulose stearoyl ester were discussed [26]. Because of its superhydrophobic properties, the material is capable of adsorbing oil from the oil/water mixture with a high selectivity. Additionally, the cellulose stearoyl ester was emulsified with an oxidized starch (OS) solution, and the resulting latex was used to impregnate commercial filter base paper, finally obtaining a hydrophobic and oleophilic membrane. This membrane has low surface energy, high strength, a long durability, and its separation efficiency was more than 99% even after ten repeated uses. As an alternative to the above material, samples based on metal meshes have some specific advantages, especially in terms of their high mechanical strength, although it is challenging to prepare metal meshes with high separation capacities. A material with interesting properties was prepared by anchoring Fe_2_O_3_ nanoclusters (Fe_2_O_3_-NCs) on a stainless steel mesh via the in situ flame synthesis method and followed by further modification with octadecyltrimethoxysilane (OTS) [27]. The oil–water separation efficiencies of the samples were found to be more than 97.5%, for all the oil/water mixtures.

An easy and cost-effective method to fabricate highly hydrophobic onion-like candle-soot (CS)-coated mesh for versatile oil/water separation, with a excellent reusability and durability was presented [28]. The sub-micron CS coating is highly hydrophobic and resistant to the harsh environmental conditions and able to efficiently separate light or heavy oil/water mixtures (>99.95%), also exhibiting high stability.

Regardless of the preparation method, the properties of the material determine its final application. However, during their use, some common problems are presented. Among these, a serious problem is erosion on the surface of various products. Polymers and their hybrid materials are suitable, in powdered form, for use as coatings in several applications. A review article [29] provides extensive information on the erosion behaviors of thermoset and thermoplastic neat resin and their hybrid material composites. This review provides information to allow researchers to explore the available selection of materials. It was noted that the thermoplastic nature of composites is a key component in determining their anti-wear properties. Finally, composites with a lower glass-transition temperature, higher ductility and greater crystallinity provide superior protection against erosion.

Highly transparent polyisocyanurate–polyurethane aerogels were synthesized, and their optical properties were studied in detail [30]. The presence and the amount of the catalyst used can influence the internal structure and the optical properties of the samples. A smaller quantity of catalyst results in smaller particles at higher transmittances.

The effect of different electrolytes on the supercapacitive properties of a nickel phthalocyanine multiwalled carbon-nanotube nanocomposite on a glassy carbon electrode (NiPcMWCNTs-GCE) was investigated [31] using electrochemical techniques. The highest specific capacitance of 6.80 F g^−1^ was achieved for the GCE-NiPcMWCNTs electrode in 5 mM [Fe(CN)_6_]^4−/3−^ prepared in 0.1 M PBS (pH 7). GCE-NiPcMWCNTs demonstrated the fastest electron-transport capability and the highest power density in H_2_SO_4_ compared to the other electrolytes. Hence, GCE-NiPcMWCNTs-modified electrodes had high stability, high energy and power densities, and a large specific capacitance.

Carbon capture, utilization, and storage (CCUS) is a way of reducing carbon emissions involving CO_2_ capture from high-emission sources. CO_2_ can be used to produce high-value-added chemicals and, on this account, covalent organic frameworks with ionic liquid-moieties (ILCOFs) have been developed as novel and efficient sorbents, catalysts or electrolytes for CO_2_ conversion. In a recent review [32], the structures and synthesis of different types of ILCOF material are presented. The application of ILCOFs for CO_2_ capture and conversion, including the reduction and cycloaddition of CO_2_, was discussed further. This interesting review presents, at the end, future directions and perspectives for ILCOFs.

Processes about the conversion of renewable and recycled C/H sources into high-value products are significant. Among these is the methanol-to-hydrocarbons (MTH) reaction, where materials like zeolites and zeotypes play a crucial role for the sustainability of the process. The synthesis, physicochemical characterization and catalytic performance of three MgAPO-11 catalysts with distinct crystal morphologies (sunflower, ball and candy) were presented in [33]. The performance of the materials was very sensitive to the shape and the crystal morphology, and it was proved that the candy-like MgAPO-11-C was superior to the other two samples. This sample exhibits the best MTH performance (4.4 g MeOH·g_cat_^−1^ h^−1^) and the best selectivity to C5+ aliphatics (64%).

Using heavy vacuum gas oil with high amounts of petroleum wax to produce high-quality liquid fuels is a very desirable process. The hydrocracking process is commonly applied, where silica-alumina catalysts are used [34]. The application of Ni W/silica-alumina catalyst in a continuous-flow fixed-bed reactor at 390, 410 and 430 °C and under the pressure of 18 MPa was discussed in view of the performance of the catalytic process. The addition of the petroleum wax to the feedstock lowers the conversion, but results in a slightly better quality of the primary liquid products. Most products prepared from the mixed feedstock had a similar or lower density and sulfur content than the products obtained from the hydrocracking of the neat HVGO. Specifically, the heavy naphtha fractions contained slightly more n-alkanes and iso-alkanes and less naphthenes and aromatics, while the middle distillates had slightly more n-alkanes, less aromatics and a cetane index higher by up to 2 units.

The pyrolysis process can be found in one other interesting application. The pyrolysis of spent plastics is a promising approach for the conversion of plastic into useful liquid fuels or other valuable materials. Metal nanoparticles supported on silica are promising because silica can stabilize the metal nanoparticles and avoid sintering and thus catalyst deactivation. Lyu et al. [35] investigated two types of core–shell mesoporous silica nanoparticles with a platinum nanocatalyst using a variety of characterization methods. The first sample had a unique amine ring structure in the middle of a shell, while the second had no ring structure. They found that the sample with the ring structure has a smaller particle size, the platinum nanoparticles are loaded evenly on the surface of the silica and the ring structure material is less stable at higher temperatures.

CO is a harmful pollutant, and much effort has been devoted to its removal, usually through its adsorption or catalytic oxidation to CO_2_ at low temperatures. To better understand the adsorption of CO on the catalyst surface, in situ and operando DRIFTS represent very powerful characterization techniques [36]. Since CO gives very intense peaks in IR, for DRIFTS studies CO(g) spectrum should be subtracted from in situ and operando DRIFTS spectra to facilitate spectral analysis. In this interesting work [36] where Pt-supported catalysts were used, it was found that a large signal of gas-phase CO overlapping with those of adsorbates is often present. Upon removing the CO(g), the carbonyls adsorbed on metallic Pt sites fully vanished in less than 10 min at 30 °C when redox supports were used, while the presence of O_2_ did not significantly affect the rate of CO decay, thus making the oxidation of CO possible with the O species from the support. In contrast, by using supports without redox properties, like alumina or titania, the O_2_ plays a significant role in CO oxidation. A broad band assigned to CO adsorbed on oxidized Pt sites, probably PtOx clusters, was stable. This suggests the route by which the physical removal of CO(g) leads to changes in the distribution of CO(ads) and a misrepresentation of the Pt site speciation, indicating the importance of operando spectroscopic techniques.

From the above outcome, it is obvious that the application of materials for the sustainability of different processes is almost unlimited. The influence of the material in one or more of the seven principles of sustainability is obvious. In the near-future, more materials with higher activity, stability and lower cost will be synthesized and applied to these already developed processes, while new tailor-made materials will be used in already existing or totally new applications.
